# Effectiveness of a Simulation-Based Education Program to Improve Novice Nurses' Clinical Judgment Skills

**DOI:** 10.7759/cureus.61685

**Published:** 2024-06-04

**Authors:** Yoshiko Kawase, Shoko Takahashi, Masako Okayasu, Yuka Hirai, Ichie Matsumoto

**Affiliations:** 1 Faculty of Nursing and Nutrition, University of Shimane, Izumo, JPN

**Keywords:** situation-based training, novice nurses, nursing education, simulation, clinical judgment

## Abstract

Introduction: We assessed the effectiveness of a simulation-based education program to improve novice nurses' clinical judgment skills.

Methods: A simulation education program was implemented for 21 novice nurses. Surveys were conducted on program satisfaction, learning, and clinical judgment skills before, immediately after, and two months after the program.

Results: Novice nurses were highly satisfied with the simulation education program. The following nine categories were identified as learnings: provide psychological care for patients, conduct sufficient observation, conduct assessment and make judgment based on observational findings, consult and report appropriately to senior nurses, take response action calmly, collect necessary information, acquire knowledge, predict patients' conditions, and make environmental arrangements. The subscale score for theoretical and practical reasoning was significantly higher immediately after and two months after the program than before it. In addition, the subscale for grasping the condition by observation was significantly higher two months after the program than before and immediately after it.

Conclusion: The novice nurses learned to sufficiently observe, obtain necessary information, and prospectively assess patients' conditions by taking part in the simulation education program. The subscale score for grasping the condition by observation was significantly higher two months after the program than before and immediately after it.

After the simulation program, novice nurses were likely actively practicing nursing; therefore, this program may not be directly responsible for the improvement of these new nurses' clinical judgment. Nevertheless, we found that the completion of the simulation program was correlated with enhanced clinical judgment.

## Introduction

Clinical judgment skills are defined as the decision to interpret and draw conclusions about a patient's needs, concerns, and health problems, as well as the decision to initiate or not initiate an action, to use or change standard methods, and to think appropriately on the spot based on the patient's response [1]. They are essential for nurses to identify abnormalities in patients, enabling them to prioritize nursing care and effectively manage multiple tasks in such situations. In Japan, novice nurses are expected to enhance their clinical judgment skills as they care for multiple patients early in their careers and make priority decisions in patient care.

Effective clinical judgment involves nurses recognizing, interpreting, and acting on cues [2]. However, previous studies on novice nurses' clinical judgment skills revealed the following: novice nurses are aware of fewer cues than experienced nurses, which may hinder their ability to identify patient problems in clinical judgment [3]; lack the correct recognition of situations and priorities [2,4]; lack the ability to identify important data [5]; and have few reasoning patterns [6]. This inexperience in clinical judgment skills affects safe patient care [7]. Novice nurses are aware of their lack of experience [8]. Better clinical judgment skills are honed through practical experience [9], but it is difficult for novice nurses who are inexperienced and lack confidence in practice to acquire accurate judgment skills in daily clinical settings.

Tanner's clinical judgment model [1] presents a framework for nurses' clinical judgment. This model comprises four phases: noticing, interpreting, responding, and reflection. Noticing is becoming aware of a clinical situation, anticipating the patient's condition, and gaining an overall understanding of the situation. Interpreting is deepening such understanding using multiple patterns of reasoning in preparation for subsequent responses. Responding is determining appropriate nursing practice based on interpretation and taking action in response. Reflection is learning through reflection on the results of interventions based on the patient's responses and applying these insights to future judgment scenarios. Furthermore, background, such as the nurse-patient relationship, interprofessional relationships, and nurses' knowledge, is considered foundational to these four phases and influences the judgment process. The clinical judgment model is a process that promotes thought patterns for effective clinical judgment. Cultivating such thoughts is crucial to develop the clinical judgment skills of novice nurses with limited knowledge from experience.

Educational approaches that are effective in enhancing the clinical judgment of nurses and nursing students include simulation, case studies, and question and answer sessions [10]. Among these, simulation-based education is an educational method to develop clinical judgment skills. Previous studies on nursing students and nurses reported the effectiveness of simulation-based education in improving their clinical judgment skills. Previous studies that examined nursing students demonstrated the effects of high-fidelity simulation [11,12] and virtual simulation [13].

Simulation-based education is also effective in improving clinical judgment skills to manage rapidly deteriorating patients [14]. Studies that examined nurses reported the effectiveness of simulation on clinical judgment in specific situations such as worsening conditions and end-of-life care [15,16]. Furthermore, studies on novice nurses that compared simulation and other educational methods, such as peer learning, reported that simulation-based education was more effective [17,18] and that debriefing promoted clinical judgment [19].

Although specific situations, types of simulations, and effects of debriefing have been studied, the effectiveness of simulation programs for training novice nurses in the thought processes that lead to clinical decisions has not been fully tested.

Among these programs, situation-based training has been reported to be effective in improving thinking, judgment, and behavior in situations similar to clinical nursing settings. However, there is no literature that has clarified the effects of situation-based training for novice nurses on clinical practice.

The purpose of this study was to create and evaluate a simulation-based education program based on a clinical judgment model [1]. The hypothesis was set as follows: the clinical judgment of novice nurses is better after the simulation program than before.

The aim was to nurture the thinking skills of novice nurses, leading to appropriate clinical judgment. We believe that the creation of this program will contribute to novice nurse education.

## Materials and methods

Study design

This study has a pre- and post-comparative and qualitative descriptive study design with content analysis.

Study period

This study was started from October 2022 to January 2023.

Selection of participants

Participants were novice nurses. The inclusion criteria for novice nurses were as follows: those who had graduated from a nurse training institution within one year and were working in a hospital ward, and working in a mid-size hospital located in one of three areas of a single prefecture in Japan that consented to participate in this study.

We invited nursing managers from hospitals employing novice nurses to participate in our study. We provided detailed information about the study's purpose, outline, significance, measures for protecting personal information, methods for data destruction, and the presentation of results at academic conferences and other events. Those who agreed to cooperate were asked to distribute a letter requesting cooperation, a statement of intent for cooperation, and a return envelope to all the novice nurses in their hospitals. The intention to participate in the study was returned by letter directly to the researcher to reduce the risk of interference by the nursing manager. Subsequently, we provided detailed written and oral explanations to the novice nurses who expressed willingness to participate, including a description of the study's purpose, outline, significance, personal information protection measures, data destruction methods, and the presentation of results at academic conferences and other events, before obtaining their consent.

Creation of a simulation-based education program

We created a simulation-based education program based on the International Nursing Association for Clinical Simulation and Learning (INACSL) Standards Committee's criteria for simulation design [20].

**Figure 1:**
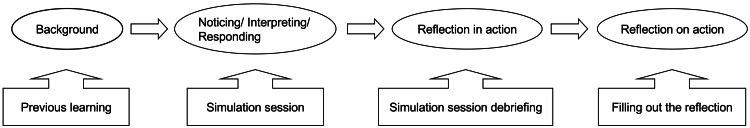
Simulation-based education flow based on the clinical judgment process in the clinical judgment model

To encourage novice nurses to think in a manner that fosters appropriate clinical judgment, we developed a simulation framework based on a clinical judgment model [1]. We structured the simulation-based education program to allow nurses to practice nursing based on this model and to perform reflection following each simulation session. Figure 1 shows the structure of the program.

**Figure 2:**
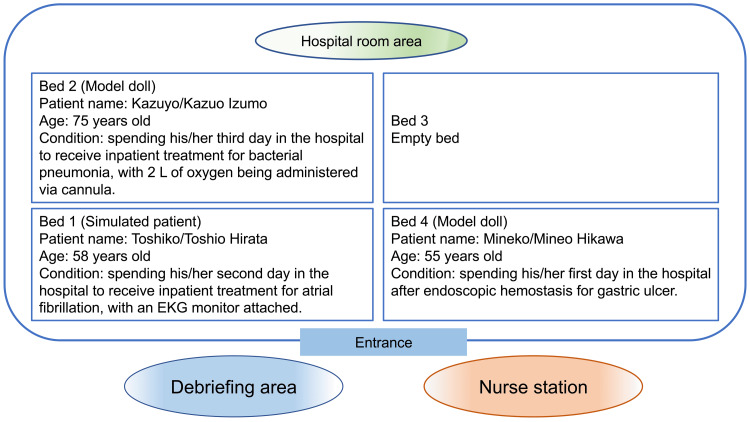
Simulated multi-bedded room EKG: electrocardiogram

A previous study [21] reported that novice nurses tend to have difficulty assessing situations when they face changes in patients' conditions or multiple tasks, which results in inadequate observations and judgments. In the present study, we discussed and developed scenarios for two situations in which a nurse is required to judge and take response action for prioritization and acute change management while taking care of multiple patients. Japanese hospitals have multi-bedded rooms that accommodate two to four patients each, in addition to private rooms. As a single nurse may be in charge of several multi-bedded rooms during a day shift, we chose a setting that simulated a multi-bedded room. Figure 2 outlines the characteristics of patients in this multi-bedded room setting. Another setting for acute change management simulated a situation where right-sided paralysis and dysarthria occur in a patient (bed 1) admitted for the treatment of atrial fibrillation.

The learning objectives of these simulations were to understand the conditions of multiple patients, to conduct temperature measurements and assessments prioritizing patient needs, and to make appropriate observations and judgments based on patients' conditions.

We created a debriefing guide based on a guide for reflection [22], using the clinical judgment model (Table 1) [1].

**Table 1:** Debriefing guide SBAR: situation, background, assessment, recommendation

Case 1		Learning objectives	(1) To be able to understand patients' conditions and situations, (2) to be able to perform temperature measurements and assessments in consideration of priorities
Clinical judgment process	Background, noticing (anticipation and initial understanding), interpreting	(1) To be able to understand patients' conditions and situations, (2) to be able to perform temperature measurements and assessments in consideration of priorities	How did you predict and understand each patient's condition from the information you collected? What are the problems with these three patients? (learners describe them on the whiteboard.)
			In what order did you perform temperature measurement and observation? In what order would you perform these procedures next time? Discuss.
			List the symptoms that Patient Hikawa complained of and what you observed. What is anemia? What do you think is the cause of his/her anemia?
			How does Hirata's atrial fibrillation affect his body?
	Responding		After observing the three patients, how do you assess each patient's condition and reflect on your findings in care?
Case 2		Learning objectives	(1) To be able to notice and understand patients' conditions and situations, (2) to be able to conduct neurological assessment appropriately and take response action, (3) to be able to report to the lead nurse in SBAR style.
Clinical judgment process	Background, noticing (anticipation and initial understanding)	(1) To be able to understand patients' conditions and situations, (2) to be able to conduct neurological assessment appropriately and take response action, (3) to be able to report to the leader nurse in an SBAR style	What did you notice about Patient Hirata's condition? What did you observe from Patient Hirata's complaints? Are there any observations that should be further examined?
	Interpreting		Regarding what you observed from Patient Hirata's complaints: after reflecting on your observation, how did you assess his/her condition? (If the assessment result is "cerebral infarction") What is cerebral infarction? How did you respond?
	Responding		How did you report to the lead nurse using SBAR? How would you do this next time?
	Reflection	Summary	Did you achieve the learning objectives? What can be applied to your future practice?

The created simulation-based education program was rehearsed with researchers using simulated patients. The program required one day to complete and consisted of a 60-minute lecture on clinical judgment, 30-minute group work, 180-minute simulation, 60-minute individual reflection, and 10-minute learning-sharing sessions.

Development of the simulation-based education program

We previously distributed case studies to the participants, instructing them to learn about the relevant diseases, treatments, symptoms, nursing care, physical assessment, and reporting in a situation-background-assessment-recommendation style. The simulations were conducted in a laboratory at the Department of Nursing, Faculty of Nursing and Nutrition, of a university. Low-fidelity simulations were performed. Each group was made up of 5-6 participants. A researcher with extensive experience in simulation-based education was assigned as a facilitator to each group. In the simulated multi-bedded room, a standardized patient played the role of a patient experiencing an acute change. All other patients were simulation mannikins. All participants experienced a simulation consisting of one of the two situations. A group debriefing session was held after each simulation, and the simulated patient provided feedback after all participants experienced the simulation. When all simulations ended, they performed individual reflections and shared their realizations and learnings with each other.

Procedure

Satisfaction With the Simulation-Based Education Program

We conducted a questionnaire survey after the completion of the program. We asked respondents about their satisfaction with the program and its usefulness in their future nursing practice using a 5-point scale (very applicable, applicable, neutral, not very applicable, and not applicable).

Assessment of Clinical Judgment Skills

We used a clinical judgment scale for Japanese nurses [23]. This scale measures clinical nurses' clinical judgment skills. Its reliability and validity have been verified.

The scale consists of two subscales, theoretical and practical reasoning and grasping the condition by observation, and 23 items, which were answered on a 5-point scale. Higher scores indicate higher clinical judgment skill levels. We assessed the novice nurses' clinical judgment skills using this scale at three points: before, immediately after, and two months after the program.

Evaluation of the Learning Effect of Simulation

We instructed the novice nurses to perform individual reflection after the simulation by filling out a reflection sheet using the REFLECT model [24]. This model consists of seven stages: Recall the events, Examine your responses, acknowledge Feelings, Learn from the experience, Explore options, Create a plan of action, and set a Timescale. We chose this model because it facilitates understanding of each stage, especially for those new to reflection.

Data analysis

Satisfaction With the Simulation-Based Education Program

We tabulated demographics and satisfaction levels.

Assessment of Clinical Judgment Skills

We calculated the mean and median total scores for each subscale before, immediately after, and two months after the program and analyzed them using the Friedman test and Bonferroni correction after the normality test, with the significance level set at 0.05.

Evaluation of the Learning Effect of Simulation

We extracted descriptions of learning from reflection sheets and analyzed them using a content analysis method [25]. We treated the entire descriptions as contextual units and individual sentences containing one content item as recorded units. We categorized and named each recorded unit based on semantic similarities. To ensure the reliability of the categories, we calculated Scott's pi [26], involving two researchers experienced in research using content analysis.

Ethical considerations

This study was approved by the Ethics Committee of the University of Shimane Izumo Campus (approval number: 356). We provided written and oral explanations of the following points to the participants to obtain their signed consent: the purpose, outline, and significance of the study; methodology; protection of human rights; data storage and processing methods; that participation was voluntary; the participants' right to withdraw or refuse to participate; no disadvantageous treatment of those who withdraw or refuse; and the publication of results in a research paper while maintaining anonymity.

## Results

Participant summary

A research request was sent to nursing administrators at 12 medium-sized hospitals, of which five hospitals agreed to cooperate. Of the 32 novice nurses working at these hospitals, 26 gave their consent to participate in the study (81% response rate).

Among the 26 participants, we included 21 who responded to the questionnaire at all of the three points in the analysis (81% response rate). The respondents' mean age was 25 years, and their demographics are shown in Table 2.

**Table 2:** Research participants' characteristics Total number of participants: 21

Characteristics	Numbers of participants (%)
Sex	
Female	19 (90.5%)
Male	2 (9.5%)
Workplace	
Internal medicine ward	11 (52.4%)
Pediatric ward	2 (9.5%)
Psychiatric ward	2 (9.5%)
Severe motor and intellectual disability ward	2 (9.5%)
Mixed ward	1 (4.8%)
Post-acute rehabilitation ward	2 (9.5%)
Other	1 (4.8%)
Final educational background	
Vocational school	18 (85.7%)
4-year university	3 (14.3%)

Satisfaction with the simulation-based education program

Ten (47.6%) respondents were very satisfied, and 11 (52.4%) were somewhat satisfied with the program. Regarding whether the program can be applied to future nursing practice, 15 (71.4%) said it was very applicable and six (28.6%) said it was somewhat applicable.

Comparison of clinical judgment subscale scores

For theoretical and practical reasoning, the median and mean scores before, immediately after, and two months after the program were 35.0 and 36.2±5.4, 46.0 and 44.0±9.0, and 47.0 and 46.2±8.6, respectively. For grasping the condition by observation, the scores were 32.0 and 33.3±4.7, 35.0 and 34.5±7.8, and 39.0 and 37.9±5.3, respectively. There were significant differences in theoretical and practical reasoning before and immediately after the program (P=0.006) and between before and two months after it (P=0.00002). There were significant differences between before and two months after the program in grasping the condition by observation (P=0.001) and between immediately after and two months after it (P=0.04) (Table 3).

**Table 3:** Comparison of clinical judgment subscale scores before, immediately after, and two months after the program ^a^P<0.05 ^b^P<0.01 ^c^P<0.001 SD: standard deviation

	Theoretical and practical reasoning			Grasping the condition by observation (N=21)		
	Before the program	After the program	2 months after the program	Before the program	After the program	2 months after the program
Mean±SD	36.2±5.4	44.0±9.0	46.2±8.6	33.3±4.7	34.5±7.8	37.9±5.3
Median	35.0^bc^	46.0^b^	47.0^c^	32.0^b^	35.0^a^	39.0^ab^

Results of content analysis of reflection sheets

The descriptions of learning extracted from reflection sheets were organized as 79 recorded units. The analysis of these units revealed nine categories summarizing the participants' learning (Table 4): provide psychological care for patients, 28%; conduct sufficient observation, 14%; conduct assessment and make judgment based on observational findings, 14%; consult and report appropriately to senior nurses, 16.5%; take response action calmly, 7.6%; collect necessary information, 6.5%; acquire knowledge, 5%; predict patients' conditions, 3.8%; and make environmental arrangements, 5%. Scott's pi was 100% for both researchers, indicating that the results were reliable.

**Table 4:** Novice nurses' learning from the simulation-based education program

Categories	Quotations	Number of recorded units (%)
Provide psychological care for patients	Approaching the patient to help alleviate some of their anxiety and worry	22 units (28%)
Consult and report appropriately to senior nurses	Immediately reporting and consult to a senior nurse when I am not confident	13 units (16.5%)
Conduct sufficient observation	Sufficiently observing the patient's condition	11 units (14%)
Conduct assessment and make judgment based on observational findings	Conducting assessment based on information including observational findings, the patient's mental status, and the patient's life background	11 units (14%)
Take response action calmly	Importance of staying calm and taking appropriate response action	6 units (7.6%)
Collect necessary information	Necessity of collecting information as a basis for appropriate judgments on patients' conditions	5 units (6.5%)
Acquire knowledge	Necessity of acquiring knowledge as a basis for appropriate judgments on patients' conditions	4 units (5%)
Make environmental arrangements	Preparing environment arrangements	4 units (5%)
Predict patients' conditions	Importance of predicting possible future events based on current diseases and symptoms	3 units (3.8%)

## Discussion

Our findings support the continued effectiveness of the simulation-based education program to improve novice nurses' clinical judgment skills.

The score for grasping the condition by observation from the clinical judgment scale was significantly higher two months after the program than before and immediately after it. The results of the content analysis suggest that the novice nurses learned to sufficiently observe and obtain necessary information and to prospectively assess patients' conditions. To make appropriate clinical judgments, it is necessary to develop patient understanding [27] and accurate insight into their conditions [1]. In the present study, the novice nurses learned the necessity of collecting information and acquiring knowledge as a basis for appropriate judgments on patients' conditions, as well as the importance of sufficiently observing such conditions and predicting possible future events based on current diseases and symptoms at all times through simulation.

The score for theoretical and practical reasoning was significantly higher immediately after and two months after the program than before it. Theoretical and practical reasoning refers to the ability to reason based on patient information and observation. Reasoning is an important phase in fully understanding situations for subsequent interventions [1]. A previous study reported that structured debriefing enhances clinical reasoning [28]. In this respect, repeated debriefings based on the clinical judgment model during each simulation session and group discussions may have improved the novice nurses' reasoning skills. Specifically, theoretical reasoning is analytical thinking based on textbook knowledge. It has been reported that novice nurses often use analytical thinking and tend to rely on textbook knowledge acquired in basic nursing education and hospital ward manuals [6]. In contrast, practical reasoning is acquired through experience. As the score for theoretical and practical reasoning significantly increased immediately after simulation, it is likely that the novice nurses used not only theoretical reasoning but also practical reasoning in clinical settings even after the simulation experience. Furthermore, by performing reflection after simulation, they may have been able to deliberate on their experience. Reflection has been shown to improve clinical judgment and clinical reasoning in complex situations [29]. We believe that reflection enabled the novice nurses to plan specific improvement measures and to use them in clinical practice.

The results of content analysis suggest that the novice nurses learned the necessity of staying calm without being hasty to make appropriate judgments, promptly consulting with senior nurses, and then asking for advice when they have difficulty in judging. It has been reported that novice nurses do not function properly due to anxiety when they encounter their first or an unexpected crisis situation [6]. In case 2, they faced a situation in which acute change management was required, as a patient suffered from right-sided paralysis and dysarthria. To make appropriate judgments in such a situation, it is important to stay calm and not to be hasty. The novice nurses seemed to have recognized the importance of staying calm and taking appropriate response action through reflection after simulation. In addition, the highest percentage of recorded units for providing psychological care for patients indicates that, during observations and assessments, attention was consistently given to the psychological needs of patients experiencing acute changes, recognizing the importance of psychological consideration for these patients.

This study demonstrated the continued effectiveness of the simulation-based education program that aimed to improve novice nurses' clinical judgment skills. However, while the effectiveness of simulation is ensured when it is conducted in a safe environment [15], simulation-based education systems are not well established in clinical settings in Japan due to insufficient human resources and locations, as well as the high cost of equipment [30]. Therefore, to continuously implement the program for novice nurses in clinical settings, the establishment of systems for novice nurse education through collaboration with educational institutions, such as universities, may be necessary.

Limitations of the study

The results of this study may be biased and limited in their generalizability because only novice nurses from medium-sized hospitals in a single prefecture were included, there was a selection bias in the departments included in this study, and there was no control group. The present study was conducted up to two months after the program was implemented; however, further studies are needed to elucidate the long-term effects on clinical judgment.

It is necessary to further verify the effectiveness of the simulation-based education program, expand its scope, and adopt a randomized controlled study design.

## Conclusions

We performed a comparison of novice nurses' clinical judgment skills before, immediately after, and two months after the simulation-based education program. We found significant increases in the score for theoretical and practical reasoning immediately after and two months after the program than before it. Furthermore, the score for grasping the condition by observation was significantly higher two months after the program than before and immediately after it. The following nine categories were identified as learnings of novice nurses: provide psychological care for patients, conduct sufficient observation, conduct assessment and make judgment based on observational findings, consult and report appropriately to senior nurses, take response action calmly, collect necessary information, acquire knowledge, predict patients' conditions, and make environmental arrangements.
